# How Healthcare Practitioners Have Supported Their Prostate Cancer Patients to Try and Overcome Barriers to Physical Activity

**DOI:** 10.3390/ijerph23070920

**Published:** 2026-07-17

**Authors:** Asmita Patel, Justin Keogh

**Affiliations:** 1 South Pacific College of Natural Medicine, Auckland 1051, New Zealand; 2 Faculty of Health Sciences and Medicine, Bond University, Gold Coast, Queensland 4229, Australia; jkeogh@bond.edu.au

**Keywords:** prostate cancer, physical activity, healthcare practitioners, survivorship, barriers, androgen deprivation therapy (ADT), physiotherapists, nurse specialists, teachable moment

## Abstract

**Highlights:**

**Public health relevance—How does this work relate to a public health issue?**

- There has been a global increase in prostate cancer incidence, especially with an aging population.
- Early detection can improve survival rates and improve quality of life in prostate cancer survivors.

**Public health significance—Why is this work of significance to public health?**

- This paper discusses why physical activity (a modifiable lifestyle factor) is important during all stages of the prostate cancer continuum.
- This paper provides practical information that healthcare practitioners and researchers can use to support physical activity in prostate cancer patients and survivors.

**Public health implications—What are the key implications or messages for practitioners, policy makers and/or researchers in public health?**

- Healthcare practitioners can have a salient role in supporting physical activity in their prostate cancer patients.
- Healthcare practitioners can provide physical activity advice and/or referral to physical activity programs or to exercise professionals, such as clinical/accredited exercise physiologists or physiotherapists.

**Abstract:**

Physical activity (PA) can provide protective benefits for prostate cancer (PCa) survivors. Healthcare practitioners are ideally positioned to promote PA to their PCa patients. This study was designed to identify how practitioners have advised and supported their PCa patients to try and overcome barriers to PA. A secondary aim was to identify if there were differences in the types of PA advice provided based on practitioner specialty and number of years in practice. Participants were 13 healthcare practitioners from Auckland, New Zealand, who provide biomedical (urology, oncology) and allied health services (physiotherapy) to men who have received a diagnosis of PCa. Participants were individually interviewed and data were analyzed using an inducive thematic approach. Three main themes and four sub-themes were identified. Physical activity advice did not appear to differ based on practitioner specialty or length of time in practice; rather, PA advice was provided to help counteract the associated side effects of specific PCa treatments. Verbal information, encouragement and resources were provided to help support PA. Specialist cancer nurses can provide long-term PA advice and support. Individualized exercise programs through physiotherapy can benefit men receiving active PCa treatment, as well as men in remission experiencing treatment-related side effects.

## 1. Introduction

Globally, prostate cancer (PCa) is one of the most diagnosed male cancers [1]. Early detection, advances in treatment options and the use of multimodal treatments have resulted in increased survivorship [2]. In the context of PCa, the terms ‘survivor’ and ‘survivorship’ consider prolonged disease course [3]. Therefore, these terms refer to both men who are in remission and are treatment-free and those who have incurable PCa and are receiving intermittent or ongoing treatment, such as hormone suppression therapy [3]. The term ‘survivor’ is part of the terminology for the cancer trajectory from prevention to end of life.

With an increase in survivorship post PCa diagnosis, a growing focus has been placed on the role that modifiable lifestyle behaviors, such as physical activity (PA), can have in improving the health-related outcomes of PCa survivors [4,5,6]. Engagement in regular PA is beneficial and safe throughout the PCa continuum, from diagnosis and treatment through to remission and survivorship, as it can provide many protective benefits [2,7,8]. Engaging in regular PA has been associated with lower prostate specific antigen (PSA) levels, a possible delay in the use of hormone suppression therapy and lower risk for PCa progression and recurrence [2,8,9]. Physical activity in the form of aerobic and resistance exercise can help counteract treatment-related side effects, especially those associated with androgen deprivation (ADT) therapy, such as abdominal weight gain, and loss of muscle and bone mass [2,5,8]. These ADT side effects can be risk factors for type 2 diabetes, osteoporosis and metabolic syndrome [2,5,8]. More than one half of PCa patients will receive ADT as a form of treatment for their PCa [10]; therefore, engagement in regular PA is important for men receiving ADT. As a group, PCa survivors are at increased risk for PCa recurrence, secondary cancers, cardiovascular disease and decreased quality of life [2,6,11,12,13]. The American College of Sports Medicine (ACSM) PA guidelines for cancer survivors recommend either 150 min of moderate-intensity PA or 75 min of vigorous PA per week [7,14]. Moderate-to-vigorous PA has been found to reduce the risk for PCa-specific mortality [15]. Engaging in regular PA during all stages of the PCa continuum (i.e., from diagnosis and treatment through to remission and survivorship) is safe for men with advanced PCa, including bone metastases [7,16].

The majority of PCa survivors are not engaging in sufficient PA to achieve health-related benefit [9,11]. A number of studies have identified and explored barriers to PA in PCa patients and survivors [17,18,19,20,21,22,23,24]. Previous studies have identified barriers that are directly related to having or having had PCa (e.g., pain and fatigue) and from the side effects of PCa treatments (e.g., urinary incontinence/leakage) [17,20,22,23,24]. Prostate cancer survivors have also reported experiencing barriers to PA that have been identified in non-cancer populations/general population, such as time constraints, pre-existing conditions, lack of motivation, previous lack of PA, increased age and lack of proximity to PA or exercise venues [17,20,22,24,25,26]. This would suggest that PCa survivors require additional assistance to perform sufficient PA for health-related benefits.

Healthcare practitioners are ideally positioned to inquire about and provide some degree of PA advice to their PCa patients, including men that are seen for surveillance and other health conditions post PCa treatment [27,28,29]. Prostate cancer survivors are receptive to receiving PA advice from their healthcare practitioners who are perceived to be trusted and credible sources of health-related information [24,27]. A number of studies have explored healthcare practitioners’ knowledge, attitudes and practices regarding PA promotion with cancer patients including PCa patients [28,30,31,32,33,34,35,36,37,38]. Earlier research carried out by our group found healthcare practitioners were more likely to provide PA advice to PCa survivors who had longer life expectancy (i.e., men in the curative group who were more likely to make a full recovery) and those at risk for chronic health conditions, as well as for men undergoing ADT to help mitigate adverse treatment-related side effects [28]. An Australian study involving 31 healthcare practitioners, the majority of whom were radiation oncologists and urologists, reported that most respondents were aware of the benefits of PA for PCa patients, especially in regard to improving quality of life (QoL) and reducing treatment-related side effects [30].

However, these practitioners infrequently provided PA advice to their PCa patients [30]. The PA advice that was provided tended to be general verbal advice, lacking detailed guidance regarding the types of PA or exercise to engage in, including intensity and frequency of activity [30]. These earlier studies have also identified barriers to PA promotion that healthcare practitioners can encounter, notably, a lack of knowledge regarding the benefits of PA during cancer continuum, concerns regarding patient safety, lack of formal training and confidence in providing PA advice, a lack of clear exercise guidelines for cancer patients, lack of resources, including referral pathways for specific PA and exercise programs, time constraints withing the consultation, and preconceptions that patients will not adhere to the PA advice provided [30,31,32,34,38].

Some healthcare practitioners, especially those who provide specialist care, have reported that provision of PA advice is not part of their role, as they provide their patients with specialized medical knowledge and information and that the provision of PA advice can be provided by other practitioners [28,30]. Limited New Zealand-based research exists that has qualitatively examined healthcare practitioners’ experiences and views regarding barriers to PA in PCa survivors. Therefore, the present study was designed to qualitatively identify and explore how practitioners have advised and supported their PCa patients to try and overcome barriers to PA, as well as identifying practitioners’ perceptions of how their advice and support could benefit their prostate cancer patients. A secondary aim was to identify if there were differences in the types of PA advice provided based on practitioner specialty and number of years in practice to provide practical insight for clinical application.

## 2. Materials and Methods

### 2.1. Study Design

The present study is a sub-study of a broader qualitative study that is composed of three sub-studies. Sixteen healthcare practitioners took part in the broader study, which comprised the following practitioner groups: three medical oncologists, one oncology nurse specialist, one radiation oncologist, four urologists, one urology nurse specialist, one radiation therapist, two general practitioners (including one who was also a Chinese medicine-trained acupuncturist), two physiotherapists, and two Chinese medicine-trained acupuncturists (including the general practitioner mentioned above). The first sub-study was designed to identify factors that influenced healthcare practitioners to either promote or not promote PA to their patients with PCa (n = 16 healthcare practitioners) [28]. The second sub-study (which is the present study) was designed to identify and explore how healthcare practitioners have advised and supported their PCa patients to try and overcome barriers to PA, as well as to identify practitioners’ perceptions of how their advice and support could benefit their PCa patients (i.e., practitioner perceptions of the benefits of PA, as well as to identify if there were differences in the types of PA advice provided based on practitioner specialty and number of years in practice (n = 13 healthcare practitioners from the broader study). The present sub-study included only 13 of the 16 healthcare practitioners from the broader study. The responses provided by two acupuncturists (one of whom was also a general practitioner) and a general practitioner were excluded from the present study. In New Zealand, acupuncturists are generally viewed by most biomedicine practitioners as being alternative practitioners and acupuncture is generally viewed by biomedicine practitioners as lacking evidence-based support in regard to supporting cancer treatment, whereas physiotherapy and physiotherapists are widely accepted practitioners in New Zealand and are perceived to provide effective PA and rehabilitation support to PCa patients and survivors. The third sub-study was designed to identify what healthcare practitioners perceived as being their PCa patients’ motives for PA and comprised five healthcare practitioners from the broader study (n = 5) [39].

### 2.2. Recruitment and Participants

Participants were recruited through purposive sampling based on the criteria that they provided specialist biomedical (oncology and urology) or allied (physiotherapy) treatment to men with PCa to gain an understanding of the scope of practice within their specific profession. Participants were recruited through Practice websites (i.e., predominately those advertising urology and oncology services). Potential participants were invited to take part in the present study via an email invitation sent by the first author, which also included a copy of the participant information sheet detailing the study. Thirty-eight email invitations were sent to obtain 13 positive responders. Those interested in taking part emailed the first author, and an interview day and time were set. Thirteen healthcare practitioners (six female and seven male) who treat men who have had a diagnosis of PCa took part in the present study. Participants were aged between 28 and 72 years of age (49.3 ± 12.0) and had been practicing in their respective fields between 9 and 31 years (23.1 ± 12.7). All participants practiced in the Auckland region of New Zealand. To have been eligible to participate in the present study, participants had to have provided specialist biomedical (i.e., oncology, urology) or allied healthcare treatment (i.e., physiotherapy) to men who had received a clinical diagnosis of PCa. Seven types of healthcare practitioners took part in the present study: medical oncologists (n = 3), a radiation oncologist (n = 1), an oncology nurse specialist (n = 1), urologists (n = 4), a urology nurse specialist (n = 1), a radiation therapist (n = 1) and physiotherapists (n = 2). The urologists and oncologists held concurrent positions at a private practice (different practices from each other) and a public hospital. Each nurse specialist worked at a different public hospital. The two physiotherapists worked in private practice (different practices from each other).

### 2.3. Interview Schedule

Members of the research team developed a single interview schedule for the broader study. The interview schedule was evidence-formed and was based on relevant literature that explored how healthcare practitioners promote PA to their PCa patients, including practitioner barriers to PA promotion. The interview schedule comprised four main sections. The first section focused on PA and contained questions that were designed to ascertain if practitioners provided PA advice, and to identify what practitioners have encountered as being their PCa patients’ barriers to PA. Questions were also designed to ascertain how practitioners have helped or advised their PCa patients to overcome barriers to PA. Practitioners were also asked what they perceived as being their PCa patients’ motives for engaging in PA. The second section of the interview schedule focused on diet and nutrition. Questions were designed to ascertain practitioners’ views on the role that diet and nutrition can have in PCa survivorship. Questions were also designed to identify whether practitioners provided any diet or nutrition information or advice to their PCa patients, and if so, to ascertain what type of information or advice was provided. Practitioners were also asked their views on the role that a low-carbohydrate healthy fat (LCHF) dietary pattern could have in PCa survivorship. The third section was designed to explore practitioners’ own PA engagement and dietary practices. The final section contained participant demographic questions. The interview schedule contained open-ended questions designed to facilitate discussion and elaboration of responses. All participants were asked the same questions in the same order.

The data presented in the present study correspond to the following question:

How have you helped or advised your patients to deal with barriers they have encountered when engaging or thinking about engaging in physical activity post-diagnosis?

### 2.4. Procedure

Participants were individually interviewed once in person by the first author at their place of work. All interviews were audiotaped and ranged in length from 13 to 35 min. All participants were asked the same questions in the same order as per the interview schedule. No written field notes were made during the interviews. If a participant’s response to a question needed to be clarified or elaborated, the first author asked the participant to clarify their response so it could be recorded for data analysis purposes. Data saturation was achieved with 16 participants, with no new themes emerging after the interview of practitioner 16. Informed written consent was obtained from each participant prior to the commencement of their interview. Ethics approval for this study was obtained from Northern A Health and Disability Ethics Committee (Reference number: 13/NTA/241/AM01). The first author is female and was employed as a Research Officer at the university at the time the study was conducted. The first author has a PhD in public health and has extensive post-doctoral experience in conducting qualitative interview-based research studies. Co-authors also hold doctorates in health disciplines and have extensive experience in conducting qualitative research. Co-authors are employed as professors at the university.

### 2.5. Data Analysis

All interviews were transcribed verbatim. Interview transcripts were manually analyzed using an inductive thematic approach based on Auerbach and Silverstein’s [40] four-step approach to thematic analysis [40]. The first step in the analysis process involved reading and re-reading each transcript for each participant response to a specific question. The second step involved identifying segments of text where participants used the same or similar words or experiences to answer the same question. The third step involved coding and naming the text, resulting in the emergence of themes. The final step involved verifying the trustworthiness of study findings and reducing individual researcher bias [40]. The first author analyzed the data and identified themes. Co-authors individually read the transcripts to ensure that participant quotes matched the themes identified. This involved verifying or disqualifying themes. Transcripts were not returned to participants prior to data analysis. The Consolidated criteria for reporting qualitative research (COREQ) reporting guidelines (File S1) were used to ensure quality reporting of the study findings [41].

## 3. Results

Three main themes and four sub-themes were identified regarding how practitioners have advised and supported their PCa patients to try and overcome barriers to PA:

Theme 1: Targeted physical activity and exercise advice for men on hormone suppression therapy.

Sub-theme 1: Verbal information regarding the health-related benefits of physical activity.

Theme 2: Verbal encouragement and support for physical activity.

Theme: 3: Implementing evidence-based physical activity support for prostate cancer survivors.

Sub-theme 1: Research evidence regarding the benefits of physical activity and exercise for prostate cancer patients and survivors.

Sub-theme 2: The need for prostate-cancer-specific exercise programs.

Sub-theme 3: The role of physiotherapy in prostate cancer survivorship.

Table 1 provides an overview of the main themes and sub-themes in relation to practitioner specialty and number of years in practice:

Each theme and sub-theme is outlined and discussed below, and quotes are provided that illustrate practitioners’ experiences and views.

### 3.1. Theme 1: Targeted Physical Activity and Exercise Advice for Men on Hormone Suppression Treatment

Oncology and urology practitioners provided PA and exercise advice to men receiving ADT. In the following quotes, oncology practitioners discussed how they advised their patients regarding the side effects of ADT and the specific types of PA and exercise they needed to engage in to help counteract treatment-related side effects, especially those relating to abdominal weight gain and loss of muscle mass and bone density. Practitioner 8 provided examples of two important types of PA that men needed to engage in daily at home to maintain their ability to carry out daily tasks of living:

“*I talk to the patients about the importance of physical exercise and managing their cancer, but also more specifically about the treatment-related side effects. With androgen deprivation therapy, I talk about change in body fat distribution and the loss of muscle in the hip and shoulder girdle. I recommend to patients that they maintain a basic level of exercise to maintain hip and shoulder strength and regular cardiovascular activity. I advise that they do two types of exercises; general fitness walking and upper and lower body resistance exercises.”*

(Practitioner 8, Medical Oncologist, number of years in practice: 12 years)

“*I would say to them as well as your regular walking, I want you to find a low chair at home and I want you to build up to three lots of ten sitting down and standing up over the day. Because I noticed that one of the things that men with prostate cancer typically loose over time is that ability to rise from a seated position, and that has impact for the toilet seat, it has impact for falls. The same with reaching up above their head. So, we talk about the washing line and saying that you need to be able to continue to do those sorts of activities of daily life. So, if you can get a couple of cans from the cupboard, do three sets of ten* [exercises] *because I think it also needs to be achievable, it needs to be something they can do in their own home that keeps them mindful about keeping those muscles because the more mindful you are about it the more likely you are to do more. But something that is easily achievable and isn’t going to dishearten them when they attempt it.*”

(Practitioner 8, Medical Oncologist, number of years in practice: 12 years)

“*We do advise them when they go onto hormone therapy that they will experience some of the side effects of hormone therapy, which is weight gain and loss of muscle and loss of bone density. So, we advise them to try and exercise to retain muscle mass and bone density and to avoid weight gain. We would recommend a combination of cardio exercise and walking. They probably wouldn’t be fit for anything else too vigorous. Walking and resistance exercise. Because another side effect of the hormone treatment can be metabolic syndrome. So, anything that is going to minimize their weight gain and maximize the muscle preservation.*”

(Practitioner 3, Oncology Nurse Specialist, number of years in practice: 31 years)

“*Patients on androgen deprivation therapy it’s known that there is muscle wasting and they can put on central weight gain. I always advise that they increase exercise. The men on hormone treatment I give some of them a brochure that actually specifies how many curls to do, how many squats to do. I would also give them more general advice like rowing or cycling for lower and rowing for upper arm exercises two or three times a week. Anything that is good for the heart is good for their prostate cancer. It’s important to have a good lifestyle, including regular exercise, control of weight, control of blood pressure, lipids and blood sugar.*”

(Practitioner 11, Radiation Oncologist, number of years in practice: 37 years)

In the following quotes, two urologists discussed how and why they provided PA and exercise advice to their PCa patients on ADT. Both urologists discussed the specific PA and exercise advice they provided, which focused on both aerobic and resistance exercises. Practitioner 2 stressed the importance of gym exercise, with the suggestion that the men approach gym staff about tailoring a specific exercise program for them. Practitioner 4 discussed how men on ADT can fatigue easily, highlighting that even though they do their best to promote PA and exercise, achieving regular PA or exercise for men with PCa can be difficult:

“*For the patients with metastatic disease which I put on ADT, I certainly do provide advice. So, I tell them about the forms of physical activity and also doing gym work and weight bearing activities. I certainly tell them that they must go to the gym, they must do weights, not only walking. Weight lifting and gym work is good for men on androgen deprivation treatment. I suggest they try and talk to one of the advisors at the gym about working out a program. I tell them about osteoporosis and muscle and also to keep their weight under control.*”

(Practitioner 2, Urologist, number of years in practice: 29 years)

“*I think it’s good for those on hormone manipulation. I try and promote it. I tell the men on hormone manipulation that they need to keep exercising. I tell them to keep physically active. But promoting it and achieving it are two different things. They probably need to be doing 30 min of aerobic exercise and preferably a little bit of resistance exercise of some sort as well. So, I will tell them that. They probably need to be doing that three times a week and preferably once a day. But one of the problems is that hormonal manipulation tends to knock your energy states on the head. You fatigue more easily.*”

(Practitioner 4, Urologist, number of years in practice: 26 years)

### 3.2. Sub-Theme 1: Verbal Information Regarding the Health-Related Benefits of Physical Activity

Several practitioners comprising urologists, an oncology nurse specialist and a physiotherapist discussed how they provided verbal information regarding the health-related benefits of PA in the context of PCa survivorship. This was especially relevant for conveying the role that PA can have in preventing certain conditions, such as cardiovascular disease and osteoporosis, and for weigh management, as well as for preventing PCa recurrence. Also discussed with PCa patients was the importance of being physically active for improved quality of life:

“*I explain to them that physical activity may extend their life. It’s also good for preventing osteoporosis. The activity is good for osteoporosis and for their muscles and to keep their weight under control.*”

(Practitioner 2, Urologist, number of years in practice: 29 years)

“*Most people with prostate cancer will die from a cardiac disease. I tell them that exercise is important for that. I think if men understood it* [physical activity] *would improve their physical well-being and also their psychological well-being, they would be better motivated to do it.*”

(Practitioner 4, Urologist, number of years in practice: 26 years)

“*If you look at the research that’s out there, we now know that exercise really plays an important part in preventing recurrent episodes of certain cancers. I always give them that information.*”

(Practitioner 10, Physiotherapist, number of years in practice: 18 years)

“*Physical activity advice for patients on hormone treatment. Any kind of exercise is going to help them from the perspective of the muscle mass and weight gain, metabolic syndrome, but also to promote their well-being and to maintain their mobility and quality of life. So that would probably be a bit more specific with the hormone patients because of all those things. To all of our patients we say, the more active they can be, the better quality of life will be. To stay active for a sense of wellbeing*”

(Practitioner 3, Oncology Nurse Specialist, number of years in practice: 31 years)

### 3.3. Theme 2: Verbal Encouragement and Support for Physical Activity

The quotes within this theme demonstrated how practitioners provided verbal encouragement for PA to their PCa patients. There was an emphasis on conveying to their patients that any activity is important and that an individual should engage in some daily PA, with the suggestion for walking as a daily activity. The focus was also on what an individual felt comfortable doing. An oncology nurse specialist highlighted how PA advice can differ based on PCa treatment type, with an example of how men receiving chemotherapy can experience more fatigue compared to men receiving other PCa treatments, and hence, it is important to tailor PA advice to avoid fatigue:

“*With the chemotherapy patients and to a certain extent, the hormone patients, fatigue. The chemotherapy can cause fatigue, and research has shown that exercise, any level of exercise that they can manage to maintain is going to counteract the fatigue to some extent. It’s all very well knowing they can overcome it with exercise, it can do them good, but you’ve got to overcome that fatigue barrier. So, we do encourage them to stay active and also for a sense of wellbeing. With the chemotherapy patients, I’ve traditionally focused more on exercise in terms of avoiding fatigue because it does counteract fatigue, because that can really debilitate. So it’s a slightly different approach for each group.*”

(Participant 3, Oncology Nurse Specialist, number of years in practice: 31 years)

“*I say to them anything you can do is a bonus and not put too much pressure on yourselves. Even if they can do little bursts through the day, just little steps. Even down to the letter box is better than sitting and doing nothing. Like this morning for example, I had a patient who said he was doing water walking and the doctor said to him that it would be really good for he could do upper arm weights as well for body strength, and I suggested perhaps he could get those weights you can wear around your hands and do that at the same time as his water walking. He’s doubling activity. So, he was responsive to that.*”

(Practitioner 3, Oncology Nurse Specialist, number of years in practice: 31 years)

“*I tell them at least go and walk every day. I try and encourage them to do whatever they feel comfortable with.*”

(Practitioner 2, Urologist, number of years in practice: 29 years)

“*I tell them to try and fit it in where they can, build it in where they can. I always say take it one day at a time and just give the advice accordingly. Every little bit counts, just get it to fit where they can.*”

(Practitioner 1 Radiation Therapist, number of years in practice: 9 years)

“*I say to the men that I have some golden fitness rules when it comes to tiredness; always rest when you need to but also try to keep up with daily activities. I do recommend that they start off with gentle walking. I tell them it can help promote or strengthen the energy that they do have.*”

(Practitioner 1, Radiation Therapist, number of years in practice: 9 years)

Written information and other resources were also provided to encourage and support PA. A urology nurse specialist discussed how she worked on a personalized PA plan with her PCa patients. This practitioner also discussed how she provided the men with contact information for local gyms. A bone health brochure comprising a fact sheet on calcium and foods to consume to support bone health for men on hormone suppression treatment (ADT) was designed by this urology nurse specialist in conjunction with dietitians at the public hospital where she works. This was provided to PCa patients receiving ADT to help mitigate the side effects of ADT regarding bone density loss. Two urologists discussed how they provided their PCa patients with a book that focused on promoting wellness after receiving a PCa diagnosis, with information covering lifestyle factors, such as diet and PA:

“*I search their local gym for them and email them the contact numbers for it. We try and settle on a personalized plan with them. When I talk with them on the phone, I say what do you do now? What would you do that’s a little step up? You’re got a dog? Do you walk it? Could you walk it twice a day instead of once a day? Could you go a little bit further? We try and personalize it.*”

(Practitioner 15, Urology Nurse Specialist, number of years in practice: 31 years)

“*For those on hormone treatment I have a bone health brochure that I give out. I’ve actually designed an information sheet on calcium, particularly, because of the bone issues with men being on hormone therapy. We actually give them a lot of suggestive foods that they might want to have. Designed with the dietitians at the hospital.*”

(Practitioner 15, Urology Nurse Specialist, number of years in practice: 31 years)

“*I often use this book of Mark Moyad as a guide and give it to my patients to read. The book covers diet and physical activity.*”

(Practitioner 2, Urologist, number of years in practice: 29 years)

“*I generally give them the Promoting Wellness book* [by Mark Moyad]*. I mainly approach that from the aspect of recovery from surgery* [radical prostatectomy]*. I’ve got someone at the moment who does a step up every morning and that patient has already lost 7 kilos.*”

(Practitioner 5, Urologist, number of years in practice: 18 years)

### 3.4. Theme 3: Implementing Evidence-Based Physical Activity Support for Prostate Cancer Survivors

This theme comprised three related sub-themes in which participants discussed their awareness of the research evidence regarding the role that PA can have within the PCa continuum, as well as the need for specific exercise programs for PCa patients and survivors. Also discussed was the role that physiotherapy could have in supporting PCa survivors engage in PA, as well as for the management of individual treatment-related side effects of PCa treatments.

### 3.5. Sub-Theme 1: Research Evidence Regarding the Benefits of Physical Activity and Exercise for Prostate Cancer Patients and Survivors

Within this sub-theme, practitioners discussed how they were aware of the research evidence regarding the role that PA and exercise can have in supporting PCa patients and survivors in relation to counteracting treatment-related side effects, in promoting well-being and for rehabilitation. A urology nurse specialist discussed that she obtained information regarding the benefits of PA and exercise by attending both international and domestic urology meetings (i.e., conferences), and by reading international guidelines regarding PA and exercise for PCa patients and survivors. A physiotherapist discussed the need for New Zealand-based PA and exercise guidelines that practitioners could use with their PCa patients. International conference attendance also provided practitioners with information regarding how other countries (i.e., neighboring Australia) provided professional support for PA and exercise. A urologist (Practitioner 16) perceived that some PCa survivors were not aware of the benefits of PA and how it could improve their quality of life:

“*I think not a lot of information is out there about the need to exercise and the need to stay healthy. Not a lot of information is out there about what happens after you have been treated for PCa. I think generally people are so grateful for being treated for the PCa and given this diagnosis that they are doing well and clear, that then nothing else gets addressed. I think that’s a huge barrier for people to realize that now that you are actually well from the cancer that there are things that we can do to improve your quality of life. So not only will you live longer, but you can enjoy that life expectancy.*”

(Practitioner 16, Urologist, number of years in practice: 16 years)

“*There is a whole raft of research into exercise cancer rehabilitation. It’s about just trying to stay relevant. I think if there were physical activity/exercise guidelines, that could be a very basic thing that someone* [other practitioners] *could suggest for them* [cancer survivors]*. I think that would help.*”

(Practitioner 10, Physiotherapist, number of years in practice: 18 years)

“*Prostate Cancer World Congress* [conference]*, the Asia Pacific one I go to every year. There is the New Zealand meeting. I always read the European and Australian guidelines. I go to conferences, particularly the Australia group, they’re very active with the exercise physiologists. I always come home very disheartened that we don’t have funded exercise physiologists for our patients here. I know that there is excellent robust evidence for the importance of exercise for mental health, prostate cancer control, as well as to try and counteract some of the side effects of the treatments.*”

(Practitioner 15, Urology Nurse Specialist, number of years in practice: 31 years)

### 3.6. Sub-Theme 2: The Need for Prostate-Cancer-Specific Exercise Programs

In the following sub-theme, Practitioner 2, a urologist, also discussed that he was aware of the benefits of PA and exercise for men with PCa by keeping up to date with research findings and through attending urology conferences. This practitioner discussed the need for specific exercise programs designed for PCa patients and survivors, which he perceived could help support their PA. This practitioner perceived that PA and exercise engagement during the PCa continuum could also benefit the men’s cardiovascular health:

“*The more I read, the more I think they may actually do best to have a formal exercise program. There was a study that recently was presented at the last urology meeting in Australia which looked at exercise and people with prostate cancer, particularly on hormone manipulation. I think we probably underutilize it* [exercise]*. It’s fine to say go and do it, but actually providing people with the means to do it may be quite poorly.*”

(Practitioner 4 Urologist, number of years in practice: 26 years)

“*I don’t deal with their exercise barriers well enough. I encourage people that physical exercise is important. Still most people with prostate cancer will die from a cardiac disease. Exercise is important for that. But again, that’s slightly different from having programs that you can put men with prostate cancer into. We need programs that you can put men with prostate cancer into. This would be the ideal situation.*”

(Practitioner 4 Urologist, number of years in practice: 26 years)

### 3.7. Sub-Theme 3: The Role of Physiotherapy in Prostate Cancer Survivorship

Within this sub-theme, a urologist and a urology nurse specialist discussed the important role that physiotherapy can have in supporting pelvic floor rehabilitation in PCa survivors. Practitioner 16 (a urologist) discussed that she worked with a physiotherapist who specialized in pelvic floor rehabilitation with males. Practitioner 15 (a urology nurse specialist) stated that there was a focus on pelvic floor exercises for men in the curative group who had urinary incontinence. Practitioner 16 discussed how she was aware of the research evidence for the effectiveness of pelvic floor rehabilitation. This urologist perceived physiotherapy to also be beneficial for general PA and exercise purposes, which in turn, was perceived to support wellbeing in PCa survivors. Within this sub-theme, the physiotherapist participants discussed what they did to support their PCa patients:

“*I do encourage them to get involved with activity. I give them general advice. If it’s someone who’s got ongoing pelvic problems, I would explain to them that they need to have pelvic rehabilitation. So pelvic floor physiotherapy so they can get better control of their pelvis or relax their pelvis. I use my model to demonstrate where the pelvis is, what the muscles are, how they work, how they don’t work. When they are working incorrectly post-surgery or post radiation, and why it’s really important that they get a physical program in terms of rehabilitating that muscle set. I work with a physiotherapist, because she does a lot of work with males with their pelvic floor and I think it’s important to have the appropriate person to deliver the appropriate care.*”

(Practitioner 16, Urologist, number of years in practice: 16 years)

“*Physiotherapy, looking at them individually as a person, working out what’s best for them. Physiotherapy it’s good for pain, often pelvic pain, urethral pain. It’s good for stress incontinence. It’s also good for activity*”

(Practitioner 16, Urologist, number of years in practice: 16 years)

“*For the curative treatment group, focus on the pelvic floor exercises for incontinence. Also, good levels of physical activity for the recovery.*”

(Practitioner 15, Urology Nurse Specialist, number of years in practice: 31 years)

“*There is a lot of research out there for pelvic floor. Physiotherapy is very good as a first step in conservative therapies. There is good evidence that pelvic floor rehabilitation is of benefit. It’s also beneficial for pain syndromes and also general wellbeing. Actually, doing some exercise is well documented in lifting people’s moods. Most of them* [her prostate cancer patients] *are pretty positive about it* [physiotherapy]. *Often, they feel really positive about it* [physiotherapy] *even if it’s not made a gross improvement on the symptoms, because I think generally doing some exercise is just good for their general wellbeing. Sometimes I have patients who are just as wet as they were when they went to see the physio* [physiotherapy], *but they feel better about it and you can see the way forward in terms of physio* [physiotherapy] *may not have been that helpful, but they can feel it does help with their general mood.*”

(Practitioner 16, Urologist, number of years in practice: 16 years)

Physiotherapy was identified as being a way to provide individualized PA programs based on what an individual required in relation to their stage of recovery from their PCa. The physiotherapists in the present study perceived physiotherapy to be beneficial for men who were inactive, as well as for the management of individual treatment-related side effects of PCa treatments. These physiotherapists also discussed how they supported the men in setting up good exercise habits so they could continue with their specific exercises at home:

“*Assessments with clients, they come in and talk about what’s going on for them. It can be any number of physical factors that have brought them to see me. It’s the effects of treatment or surgery that they are coming to see me for. Then we would do hands on physical or manual therapy as well if required, and then always branch off into exercise after that. So individualized treatment plans. Everybody is different. There is definitely no one size fits all. There are one on one individual assessments. It’s really important to see what the client needs at that particular stage of their recovery.*”

(Practitioner 10, Physiotherapist, number of years in practice: 18 years)

“*We have individualized programs. If someone is very new to exercise, we just take a very gentle approach with them. We definitely don’t want to over exercise them because they will just be turned off completely. You have to pace them and structure them for their program. Even just getting them to go for a walk to the end of the drive and back. That might be where we start off.*”

(Practitioner 10, Physiotherapist, number of years in practice: 18 years)

“*I see them at all times during the spectrum. That’s why it is very individualized. When they are out of* [completed] *their treatment, the focus is setting them up with good exercise habits to be able to continue on with and carry on with their exercises. I run a group and have classes here as well as for cancer rehabilitation.*”

(Practitioner 10, Physiotherapist, number of years in practice: 18 years)

“*I’ll cover everything, like they’re going to have to wear pads. We’ll do an assessment, get them started doing exercises. We focus on pelvic floor exercises. They’re not with us every day. The main thing is doing them* [the exercises] *at home as well.*”

(Practitioner 9, Physiotherapist, number of years in practice: 21 years)

## 4. Discussion

In the present study, PA advice did not appear to differ based on practitioner specialty or length of time in practice. The healthcare practitioners who took part in the present study provided PA advice to their PCa patients based on the type of PCa treatment(s) an individual received in relation to focusing on how to counteract the associated side effects of the specific treatment(s); this was especially evident in relation to practitioners who administered ADT. All practitioners who took part in the present study perceived PA to be beneficial for their PCa patients.

The oncology and urology practitioners who administered ADT discussed how they provided the men with information regarding the side effects they would experience when undergoing ADT treatment. The practitioners from these two different specialties discussed how they provided targeted PA advice for men undergoing ADT to help counteract treatment-related side effects, such as muscle loss and weight gain, which are risk factors for type 2 diabetes and metabolic syndrome [2,5,8]. The practitioners in the present study provided detailed information regarding the specific types of PA and exercise (i.e., resistance and aerobic), including the frequency and intensity that men undergoing ADT needed to engage in. A medical oncologist provided targeted PA and exercise advice to ensure that men would maintain their hip and shoulder strength to be able to continue to engage in activities of daily living. In line with our study findings, oncology nurses (from both Australia and New Zealand) also perceived that PA was beneficial in improving the activities of daily living [31]. Both a scoping review [24] and a systematic review [42] designed to identify facilitators and barriers to PA in men with PCa, including those receiving ADT, reported that men wanted to receive PA advice from their healthcare practitioners who were perceived to be an important, trusted source of lifestyle information [24,42]. Receiving PA advice or recommendations from a healthcare practitioner can act as a motive for engaging in PA for men with PCa [24].

Verbal information regarding the health-related benefits of PA in relation to PCa survivorship was provided by a range of practitioner specialties in the present study. This information focused on the preventive role that PA can have in minimizing risk factors for cardiovascular disease, osteoporosis and obesity, for preventing PCa recurrence and for improving quality of life. This verbal information imparted by practitioners in alignment with men’s willingness to adopt healthier lifestyles (i.e., engaging in regular PA, making healthier dietary changes) can help facilitate a ‘teachable moment’ when men are more receptive to making positive lifestyle changes [27,43]. A UK-based qualitative study [27] designed to identify and explore the views and experiences of healthcare practitioners (n = 10, urological surgeons, oncology nurse specialists, physiotherapist), men with localized PCa (n = 16), and their partners (n = 7) regarding the provision of diet and PA advice post PCa diagnosis and treatment also reported similar findings [27]. Healthcare practitioners in the Sutton study [27] were aware of the ‘teachable moment’ opportunity, with some providing lifestyle (i.e., dietary and PA) advice at the time of diagnosis, while other practitioners thought this advice was best provided post-treatment as men had to deal with their diagnosis and treatment [27]. However, the men and their partners would have preferred to have received lifestyle information at the time of diagnosis [27]. Lifestyle information/advice provided at diagnosis can result in men feeling empowered if they engage in behavior (i.e., regular PA) that can benefit their recovery, and likewise, help prevent PCa recurrence post-treatment [27].

The urology practitioners in the present study also provided written information and other resources to encourage and support PA. This included providing PCa patients with a book that focused on promoting wellness post-diagnosis, which included information on lifestyle factors relating to diet and PA. The role that nurse specialists can play in providing support during the PCa continuum was evident in the present study. A urology nurse specialist discussed how she worked on a personalized PA plan with PCa patients, predominantly through phone consultations. This nurse specialist also provided phone numbers for local gyms that men could attend. In addition to a personalized PA plan, this practitioner also designed a bone health brochure with dietitians at the hospital where she worked. This brochure contained information regarding food that men receiving ADT should consume to support their bone health. In line with our findings, research carried out in Australia, Canada and the United Kingdom has reported on the salient role that nurse specialists can have in helping meet the unmet support care needs of PCa patients and survivors, including those receiving long-term care, especially in the context of lifestyle advice, such as PA information and support [44,45,46,47]. An Australian nurse-led telephone-based psychoeducation program involving 20 PCa patients receiving ADT, conducted over 10-weeks and comprising of four phone sessions provided by a nurse specialist, was perceived to be acceptable [44]. The PCa patients in the Sara et al. study [44] found the telephone-based intervention helpful in relation to the provision of knowledge regarding the impact that ADT can have on different aspects of their physical and psychological well-being. The intervention also helped improve lifestyle behaviors pertaining to PA and diet [44].

In the present study, several practitioners (urology practitioners and a physiotherapist) discussed that they were aware of the research evidence regarding the benefits of PA throughout the PCa continuum. This information was obtained through attending international and domestic urology meetings (i.e., conferences) and by keeping up to date with research findings and by reading international guidelines for PA and exercise for PCa patients and survivors. A physiotherapist also discussed the need for New Zealand-based PA and exercise guidelines that practitioners could refer to. Two urology practitioners (a urologist and a urology nurse specialist) discussed how international conference attendance provided them with knowledge regarding how other countries provided PCa patients and survivors with practical support for PA. This led to a urologist discussing the need for a formal PCa-specific exercise program that he perceived would help support men to be physically active. The present findings emphasize the importance of New Zealand healthcare practitioners keeping up to date with current evidence-based international PA and exercise guidelines and supportive care resources for PCa, which could also be potentially used as models to serve as a foundation for the development of New Zealand-based programs and referral pathways for PCa patients. The development of a centralized list of these resources and programs for New Zealand PCa patients and survivors could be helpful in ensuring that men are receiving the PA and dietary information they need.

Urology practitioners and physiotherapists in the present study discussed how physiotherapy could help initiate and support PA in PCa patients and survivors, as physiotherapists could provide individualized PA programs. Individualized programs were perceived by both urology practitioners and physiotherapists as being able to benefit men who were inactive, as well as to help manage individual treatment-related side effects of PCa treatments. The use of physiotherapy to support PCa patients prior to PCa surgery or during active treatment and post-treatment is well documented in the research literature [48,49,50]. Urologists and physiotherapists in the present study were aware of the research evidence regarding the benefits of pelvic floor rehabilitation for PCa survivors. Physiotherapy can benefit men experiencing urinary incontinence and erectile dysfunction post PCa treatment through targeted pelvic floor exercises [48,50]. Likewise, men receiving ADT can benefit from physiotherapist-initiated exercise designed to help counteract loss of muscle mass and the associated loss of muscle strength and physical function [47]. Individualized exercise guidance provided by a physiotherapist has been well received by men undergoing ADT [51].

A qualitative interview-based approach enabled practitioners to discuss in detail the various ways in which they have helped their PCa patients try and overcome barriers to PA. Seven types of healthcare practitioner specialties (inclusive of the allied health physiotherapists) were interviewed, hence providing more insight regarding the types of PA and exercise advice and support that are provided by different practitioner specialists who may interact with PCa survivors. This also provided more insight into how different practitioner groups may help their PCa patients try and overcome barriers to PA. The range of healthcare practitioners could be considered a strength, in that it indicates a multidisciplinary approach, whereby a range of healthcare practitioners can all play a role in helping to support the health and well-being of men with PCa. There were also study limitations. All but one participant self-selected to participate in this study. All practitioners conveyed that they provided PA advice, and all participants perceived PA to be beneficial throughout the PCa continuum. Another limitation of this study is that the outcomes of the practitioner advice and support were not measured, and the practitioners did not convey if their advice did help their PCa patients to engage in PA. The small sample size may limit the transferability of study findings to similar practitioner groups, as 13 practitioners may not represent the majority of providers. It must also be highlighted that these providers had similar outlooks regarding PA despite being from different practitioner groups. Likewise, the limitation is that these providers responded to a call for participant recruitment and were likely advocates for PA and exercise. Study findings can be utilized by different groups of healthcare practitioners (i.e., urologists, oncologists, nurse specialists, primary care physicians), including allied practitioners, such as physiotherapists, exercise physiologists and researchers to support PCa patients and survivors to engage in PA during the PCa continuum (from diagnosis through to remission and survivorship). The present study identified that there was a lack of referral pathways for PCa-specific PA and exercise programs in New Zealand. Therefore, there needs to be a focus on implementing programs that can support PCa survivors to engage in PA.

## 5. Conclusions

Physical activity advice did not appear to differ based on practitioner specialty or length of time in practice. The PA advice that was provided by practitioners in the present study was similar (irrespective of practitioner specialty), as it was targeted to help counteract the side effects of specific PCa treatments (i.e., the side effects of ADT, pelvic rehabilitation). Somewhat different to previous studies in this area, all practitioners who took part in this study conveyed that they provided PA advice to their PCa patients, and all practitioners perceived that the PA advice, support and resources they provided were beneficial to their PCa patients. There was a variation in the level of support provided by different practitioners. Some offered more general advice, while others, such as nurse specialists, offered more personalized advice. This suggests that while many practitioners may provide PA and dietary advice, the depth, specificity, and degree of individualized support may differ considerably. Prostate cancer nurse specialists were identified as being able to provide ongoing (long-term) support during the PCa continuum, especially relating to lifestyle information that focused on PA. While the present study identified that there was a lack of referral pathways for PCa-specific PA and exercise programs in New Zealand, physiotherapy was identified as a way to help initiate and support PA in men through the provision of individualized exercise programs. Future research will identify what practitioners perceive as being their PCa patients’ motives for PA.

## Figures and Tables

**Table 1:** Overview of the main themes and sub-themes in relation to practitioner specialty and number of years in practice.

Themes and Sub-Themes	Practitioners Who Provided Responses Under Each Theme or Sub-Theme
Theme 1: Targeted physical activity and exercise advice for men on hormone suppression therapy	Medical Oncologist, Practitioner 8 (12 yrs in practice) (two quotes under this theme) Oncology Nurse Specialist, Practitioner 3 (31 yrs in practice) Radiation Oncologist, Practitioner 11 (37 yrs in practice) Urologist, Practitioner 2 (29 yrs in practice) Urologist, Practitioner 4 (26 yrs in practice)
Sub-theme 1: Verbal information regarding the health-related benefits of physical activity	Urologist, Practitioner 2 (29 yrs in practice) Urologist, Practitioner 4 (26 yrs in practice) Physiotherapist, Practitioner 10 (18 yrs in practice) Oncology Nurse Specialist, Practitioner 3 (31 yrs in practice)
Theme 2: Verbal encouragement and support for physical activity	Oncology Nurse Specialist, Practitioner 3 (31 yrs in practice) (two quotes within this theme) Urologist, Practitioner 2 (29 yrs in practice) (two quotes within this theme) Radiation Therapist, Practitioner 1 (9 yrs in practice) (two quotes within this theme) Urology Nurse Specialist, Practitioner 15 (31 yrs in practice) (two quotes within this theme) Urologist, Practitioner 5 (18 yrs in practice)
Theme 3: Implementing evidence-based physical activity support for prostate cancer survivors Sub-theme 1: Research evidence regarding the benefits of physical activity and exercise for prostate cancer patients and survivors Sub-theme 2: The need for prostate-cancer-specific exercise programs Sub-theme 3: The role of physiotherapy in prostate cancer survivorship	Urologist, Practitioner 16, (16 yrs in practice) Physiotherapist, Practitioner 10 (18 yrs in practice) Urology Nurse Specialist, Practitioner 15 (31 yrs in practice) Urologist, Practitioner 4 (26 yrs in practice) (two quotes under this sub-theme) Urologist, Practitioner 16 (16 yrs in practice) (three quotes under this sub-theme) Urology Nurse Specialist, Practitioner 15 (31 yrs in practice) Physiotherapist, Practitioner 10 (18 yrs in practice) (three quotes under this sub-theme) Physiotherapist, Practitioner 9 (21 yrs in practice)

## Data Availability

Data cannot be shared openly due to restrictions of ethics approval and the anonymization of participants.

## Supplementary Materials

The following supporting information can be downloaded at: https://www.mdpi.com/article/10.3390/ijerph23070920/s1, File S1: COREQ (COnsolidated criteria for REporting Qualitative research) Checklist.

## Abbreviations

The following abbreviations are used in this manuscript:

PA	Physical activity
PCa	Prostate cancer
ADT	Androgen deprivation therapy
PSA	Prostate specific antigen
